# Perspectives of Early to Mid-Career health professionals: Future public health challenges and opportunities in the Asia-Pacific region

**DOI:** 10.1371/journal.pgph.0006145

**Published:** 2026-04-02

**Authors:** Claudia Rouse, Joshua Aliifaalogo, Gemma Allinson, Kiran Bam, Dylan D. Barth, Dinesh Bhandari, Ryan Brigham, Matthew Carvalho, Sian Dooley, Seamus Horan, Bethany Howard, Clare Humphreys, Sarah Lang, Samuel McEwen, Rob Mitchell, Chowa Nkonde, Lydia O’Meara, Lanique Naolina Pitasua, Rebecca Reilly, Adeel Shareef, Juliana Silva, Wenbo Song, Stefanie Vaccher, Soazic Elise Wang Sonne, Arporn Wangwiwatsin, Chaturangi Yapa, Xenia Zubenko, Dragan Ilic

**Affiliations:** 1 School of Public Health and Preventative Medicine, Monash University, Melbourne, Victoria, Australia; 2 Council of Academic Public Health Institutions Australasia (CAPHIA), Lyons, Australian Capital Territory, Australia; 3 St Vincent’s Hospital, Melbourne, Victoria, Australia; 4 Moana Connect, Auckland, New Zealand; 5 School of Public Health, University of Queensland, Herston, Queensland, Australia; 6 Department of Medicine, School of Clinical Sciences at Monash Health, Monash University, Clayton, Victoria, Australia; 7 Wesfarmers Centre of Vaccines and Infectious Diseases, The Kids Research Institute Australia, Nedlands, Western Australia, Australia; 8 Health in Focus, Melbourne, Victoria, Australia; 9 Sunway Centre for Planetary Health, Sunway University, Kuala Lumpur, Malaysia; 10 O’Neill Institute for National and Global Health Law, Georgetown University, Washington, District of Columbia, United States of America; 11 School of Primary & Allied Health Care, Monash University, Melbourne, Victoria, Australia; 12 The Royal Melbourne Hospital, Melbourne, Victoria, Australia; 13 School of Population Health, University of New South Wales, Sydney, New South Wales, Australia; 14 Victorian Heart Institute, Monash University, Clayton, Victoria, Australia; 15 Monash Centre for Health Research and Implementation, School of Clinical Sciences, Monash University, Clayton, Victoria, Australia; 16 Burnet Institute, Melbourne, Victoria, Australia; 17 Acute and Critical Care Division, Monash University, Melbourne, Victoria, Australia; 18 Emergency & Trauma Centre, Alfred Health, Melbourne, Victoria, Australia; 19 The Institute of Tropical Medicine and International Health of Charité – Universitätsmedizin, Berlin, Germany; 20 Natural Resources Institute, University of Greenwich, Chatham Maritime, Kent, United Kingdom; 21 Independent Consultant, Food and Agriculture Organization of the United Nations, Dili, Timor-Leste; 22 The Australian National University, Canberra, Australian Capital Territory, Australia; 23 Intensive Care Unit, Royal Melbourne Hospital, Melbourne, Victoria, Australia; 24 Australian and New Zealand Intensive Care Society (ANZICS), Prahran, Victoria, Australia; 25 GIN Discovery Program, Monash University, Melbourne, Victoria, Australia; 26 Department of Non-Communicable Disease Epidemiology, London School of Hygiene & Tropical Medicine, London, United Kingdom; 27 School of Tropical Medicine and Global Health, Nagasaki University, Nagasaki, Japan; 28 Health, Nutrition and Population Department, World Bank Group, Washington, District of Columbia, United States of America; 29 Department of Systems Biosciences and Computational Medicine, Faculty of Medicine, Khon Kaen University, Khon Kaen, Thailand; 30 Cholangiocarcinoma Research Institute, Khon Kaen University, Khon Kaen, Thailand; 31 South-East Public Health Unit, Monash Health, Melbourne, Victoria, Australia; PLOS: Public Library of Science, UNITED STATES OF AMERICA

## Emerging leaders for public health

Contemporary public health challenges, such as climate change, directly influence the work of early-mid career (EMC) health professionals and senior health students [1]. Despite this intersection, little research exists to examine how the views and experience of emerging leaders can assist with capacity building in health [1]. Emerging leaders, including health professionals and senior post-graduate health students, share unique visions for the future of health and are central to shaping this future. They contribute the largest proportion of the health workforce and consequently play a pivotal role in shaping healthcare research and reform, particularly in the midst of climate trends, geopolitics, economics and emerging pathogens [1].

Providing a platform for emerging leaders fosters a community of practice to share resources, knowledge and education often beyond discipline and geographic boundaries [2]. It also facilitates contributions to quality primary literature in early careers [2]. Consequently, including emerging leaders in agenda setting is crucial to primary health care, international universal health coverage and achieving sustainable development goals (SDGs) [3].

This article serves to contribute to literature outlining the challenges, opportunities and engagement of emerging leadership in healthcare. It aims to move beyond well-documented challenges in public health to propose actionable, region-specific reforms grounded in lived and professional experience. In doing so, this article formalises the reflections from 26 EMCs selected across the Asia-Pacific region and beyond at the World Health Summit (WHS), Melbourne 2024 [4]. Each EMC provided two written reflections on sessions attended as well as perspectives on the future of public health in Asia-Pacific. EMC’s professional backgrounds ranged from health research, policy, allied health and medicine.

### Perspective *of* emerging leaders

#### Challenges to public and global health.

Emerging leaders identified several persistent challenges to public and global health including incorporating health equity, climate change, research translation and health workforce sustainability. Some systems fail to fully address gender, health literacy, financial disparities, First Nations Peoples in Asia-Pacific and diverse communities. Climate change is a contributor to these discrepancies with implications on population displacement and physical and mental wellbeing particularly for nations in Asia Pacific on the forefront of climate mediated effects. There is an urgent need to prioritise the transition between research and implementation and a sustainable health workforce. Additionally, health workforce shortages, particularly for low- and middle-income countries (LMIC) in the Asia-Pacific are exacerbated by non-circular migration and extended training periods.

#### Opportunities and Key priorities for fostering public and global health.

Key opportunities for public and global health include co-design approaches, multidisciplinary collaboration, digital solutions and efficient resource allocation. Co-design and diverse teams utilising local innovation, lived experience and Indigenous knowledge can underpin longevity of solutions and meaningful change. To achieve this, prioritising equitable health financing/staffing, governance and digital solutions to safeguard public health in the face of pandemics, climate disasters and public health threats is required.

Digital solutions such as generative artificial intelligence (AI) serve as a unique and powerful approach to advancing healthcare technologies. Application to marginalised communities should be cautioned and regulation actioned to consider the representation of AI derived learnings and intellectual property, however. The World Health Organisation’s (WHO) AI ethics and governance guidance for large multi modal models (LMMs) offers practical guardrails that can be adapted to Asia–Pacific contexts.

#### Engaging emerging leaders in public and global health.

Representation of EMCs in decision-making processes can still be improved. A proactive call to action for diversity of voices and representation on issues pertinent to the EMC workforce is needed. Collaborative opportunities and mentorship to build capacity across research, education, policy and advocacy present as an enormous opportunity to leverage the potential of EMC professionals.

## Discussion

Key themes outlined by EMCs align with the SDGs and regional priorities, which have set the agenda for public health and provide a framework for coordinated efforts to address determinants of health [5]. We contextualise the findings within the existing literature and suggest improvements to region specific reforms within four key action areas.

Action 1: Targeting the social determinants of health through policy adoption and co-design, is a key alignment. Community representation and co-design improve health outcomes and empowerment by incorporating local knowledge and participation. This directly translates to many aims outlined in SDG 10 (reducing inequality), SDG 17 (partnership for the goals) and the Australian Enhanced Consumer Engagement Framework [5,6]. Despite co-design emerging as a preferred collaborative approach, lack of consistency in application limits its efficacy. More transparent co-design principles with early-stage engagement of community and recognised models are therefore needed. This is a matter of urgency for climate vulnerable communities and data sovereignty [7].

Action 2: Adaptive capacity to respond and prevent climate related hazards is also a focus [4]. The unique position of healthcare systems as a contributor to emissions as well as a recipient of the indirect detrimental climate effects, stresses this urgency. Utilising a ‘bottom up’ approach to engage communities most affected by climate change demonstrates an important avenue to engage those often dismissed by higher-level objectives [8]. EMCs suggested unique avenues including recognising healthcare’s own direct impact on climate change by considering MBS/PBS applications to report emissions. Despite well recognised approaches, ongoing climate mitigated effects in healthcare, likely reflect public financial management bottleneck for health service delivery and response [8].

Action 3: Workforce sustainability across Asia-Pacific could be enhanced through adequate skills exchange, regionally coordinated training and clinician led reform. Non-circular migration is an increasing regional challenge. For example, in Fiji one third of nursing positions remain vacant largely due to continued migration abroad [9]. Climate trends and geo-political stressors contribute a large and persisting gap between current and estimated workforce requirements. An increasing focus on reciprocity in health workforce training, information communication technology, and clinical led reform can improve efficiencies, retention and sustainable healthcare [10].

Action 4: The engagement of emerging leaders provides a means to enhance the future of global public health and achievement of SDG targets [11,12]. For example, EMCs can uniquely navigate use cases, regulation and data bias within artificial intelligence models in healthcare given they this technology is uniquely positioned do advance healthcare in their careers [13]. Mentorship also provides a sustainable mechanism for engagement such as in the Asia-Pacific Academic Mentoring Program [1,14]. Mentorship programs could be incorporated into internship/graduate position programs, accreditation practices and expanded to broader events such as WHS. Mentorship programs in healthcare facilities and educational institutions also require avenues for investment, structured programs and goals [1].

Our findings support growing recognition that healthcare education, training programs and workplaces have a vested interest in supporting emerging leader research engagement and translation, training, skill development and retention [3,11]. This places responsibility on universities and accreditation practices to account for public health challenges and opportunities, regularly reviewing programs and fostering effective adaptable leaders in program design [15]. Protected training and allocated investment is important to securing a science literate workforce and adoption of future evidence based practices in all key action areas.

## Conclusions

This paper gives a voice to EMCs in health discussions and planning. We highlight important public health themes including climate change, workforce sustainability and research translation. Key action areas are synthesised against considerations identified by EMC professionals and key solutions brought forward. Our findings contribute to growing literature and important discussions around emerging leader involvements in higher level healthcare decisions.

## Supporting information

S1 Appendix World Health Summit Regional Meeting Melbourne 2024 Session Titles. (DOCX)

## Abbreviations

WHS: World Health Summit
WHO: World Health Organization
SDG: Sustainable Development Goal
EMC: Early mid-career
LMIC: Low- and middle-income country

## References

[1] Wong BLH, Siepmann I, Chen TT, Fisher S, Weitzel TS, Nathan NL, et al. Rebuilding to shape a better future: the role of young professionals in the public health workforce. Hum Resour Health. 2021;19(1):82. doi: 10.1186/s12960-021-00627-7 34256785 PMC8276547

[2] Krauss A, Danús L, Sales-Pardo M. Early-career factors largely determine the future impact of prominent researchers: evidence across eight scientific fields. Sci Rep. 2023;13(1):18794. doi: 10.1038/s41598-023-46050-x 37914796 PMC10620415

[3] World Health Organization. Health workforce requirements for universal health coverage and the sustainable development goals. World Health Organization website. 2016. https://iris.who.int/bitstream/handle/10665/250330/9789241511407-?sequence=1

[4] World Health Summit 2024. World Health Summit website. Accessed 2024 November 10. https://www.worldhealthsummit.org/

[5] United Nations. The 17 Goals. Accessed 2024 October 20. https://sdgs.un.org/goals

[6] Butler T, Gall A, Garvey G, Ngampromwongse K, Hector D, Turnbull S, et al. A Comprehensive Review of Optimal Approaches to Co-Design in Health with First Nations Australians. Int J Environ Res Public Health. 2022;19(23):16166. doi: 10.3390/ijerph192316166 36498237 PMC9735487

[7] World Health Organization. For the future: towards the healthiest and safest region. World Health Organization website. 2020. Accessed 2024 October 25. https://www.who.int/publications/i/item/WPR-2020-RDO-001

[8] Ansah EW, Amoadu M, Obeng P, Sarfo JO. Health systems response to climate change adaptation: a scoping review of global evidence. BMC Public Health. 2024;24(1):2015. doi: 10.1186/s12889-024-19459-w 39075368 PMC11285469

[9] Pacific Heads of Nursing and Midwifery. Pacific Heads of Nursing and Midwifery Meeting. Pacific Community Public Health Division. 2025. https://phd.spc.int/sites/default/files/p-related-files/2025-03/2025%20PHoNM%20IP%202.5_Nursing%20migration%20study%20in%20Fiji.pdf

[10] Department of Public Health B-BU. Round table: Circular migration of the health and care workforce: how can we design sustainable policies?. Eur J Public Health. 2023;33(Suppl 2):ckad160.537. doi: 10.1093/eurpub/ckad160.537

[11] Augsberger A, Young A, Toraif N, Morris M, Barnett KG. Youth engagement to achieve health equity: Are healthcare organizations and leaders prepared? Am J Community Psychol. 2023;71(3-4):410–22. doi: 10.1002/ajcp.12656 36661430

[12] Stanford FC. The Importance of Diversity and Inclusion in the Healthcare Workforce. J Natl Med Assoc. 2020;112(3):247–9. doi: 10.1016/j.jnma.2020.03.014 32336480 PMC7387183

[13] Kotp MH, Ismail HA, Basyouny HAA, Aly MA, Hendy A, Nashwan AJ, et al. Empowering nurse leaders: readiness for AI integration and the perceived benefits of predictive analytics. BMC Nurs. 2025;24(1):56. doi: 10.1186/s12912-024-02653-x 39819624 PMC11737245

[14] International Science Council. Asia-Pacific Academic Mentoring Program. 2023. Accessed 2024 December 12. https://council.science/our-work/asia-pacific-academic-mentoring-program/

[15] Rizvi DS. Health education and global health: Practices, applications, and future research. J Educ Health Promot. 2022;11:262. doi: 10.4103/jehp.jehp_218_22 36325224 PMC9621358

