# Supplementary material for: Perspectives of Early to Mid-Career health professionals: Future public health challenges and opportunities in the Asia-Pacific region
Source: PLOS Glob Public Health. 2026 Apr 2;6(4):e0006145. doi: 10.1371/journal.pgph.0006145 (PMC13046134; doi:10.1371/journal.pgph.0006145)
Supplement: S1 Appendix — (DOCX) [file pgph.0006145.s001.docx]

**APPENDIX 1.** World Health Summit Regional Meeting Melbourne 2024 Session Titles

| Plenaries   - From Climate Change Evidence to Action: Accelerating pathways for healthy people and a healthy planet - Geopolitics and Health: Achieving equity in a divided world - Shaping the future of health across Asia and the Pacific - Thriving Communities: Priorities for living well and living well together   Concurrent Sessions   - Achieving health equity for women and girls - A One Health approach to public health: a call to action - Building capacity of healthcare workers providing care to refugees and migrants - Childhood obesity prevention: innovative food policy and setting-based approaches from Asia and the Pacific. - CKM syndrome in the Asia Pacific: tackling this highly prevalent condition with an equity focus - Climate change and health of First Peoples - Clinician led health system reform: benefits for patients, payers, policy and practitioners - Emerging and reemerging infectious health threats: opportunities for effective regional collaboration and leadership - From climate change evidence to action: accelerating pathways for healthy people and a healthy planet - Health and human rights: IAS Lancet Commission focusing action in 2024 - Improving health by strengthening healthcare financing in Asia Pacific - Is the world ready for the next pandemic? - Lived and living experience guiding mental health reform - Living and breathing health - Net zero and climate resilient health services: how do we get there? - New and old knowledges: First Peoples health and AI - Pacific priorities for prevention of violence against women - Priorities for education, training and a future proof workforce - Rethinking drug policy: minimising harm and unintended consequences - The power of finance and investments for addressing the commercial determinants of health - Trans-continental collaboration for global health - Where do the real leadership opportunities lie for SDGs and health |
| --- |
